# Supplementary material for: Yiai Fuzheng decoction inhibits triple-negative breast cancer by remodeling the immune microenvironment
Source: Front Immunol. 2025 Sep 30;16:1615631. doi: 10.3389/fimmu.2025.1615631 (PMC12518410; doi:10.3389/fimmu.2025.1615631)
Supplement: Supplementary file 2 [file DataSheet1.zip › Supplementary File 2/Treatment/C_vs_H/07_Pathway_Analysis/Pathway_Analysis/Sig_Pathways_ID/Lysine degradation.pdf]

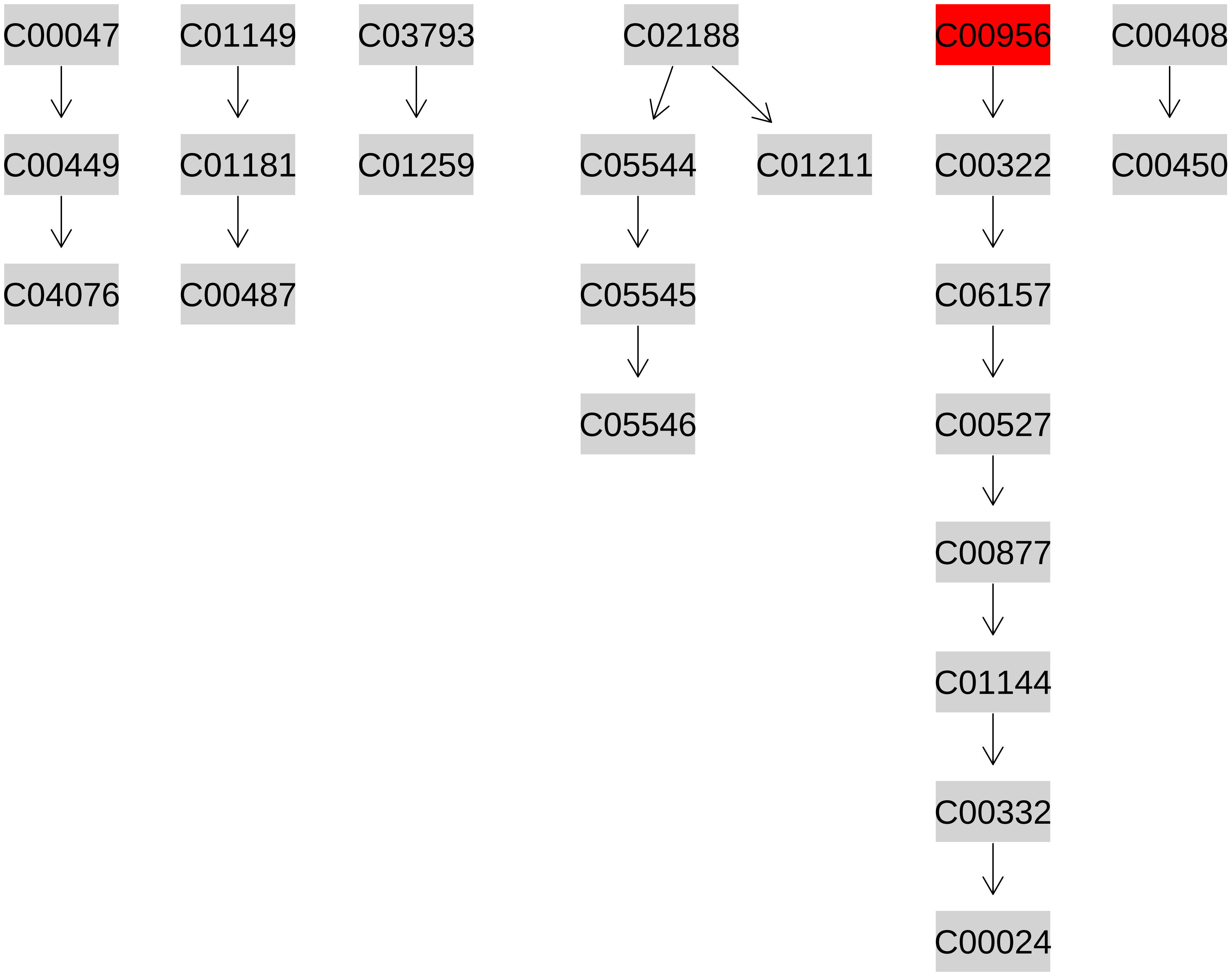
C00047
C00449
C04076
C01149
C01181
C00487
C03793
C01259
C02188
C05544
C05545
C05546
C01211
C00956
C00322
C06157
C00527
C00877
C01144
C00332
C00024
C00408
C00450
